# First person – Louis Widom

**DOI:** 10.1242/bio.062513

**Published:** 2026-02-24

## Abstract

First Person is a series of interviews with the first authors of a selection of papers published in Biology Open, helping researchers promote themselves alongside their papers. Louis Widom is first author on ‘ Antibiotics modulate *Escherichia coli*-derived bacterial extracellular vesicle production and their upregulation of ICAM-1 in human endothelial cells’, published in BiO. Louis is a PhD candidate in the lab of Thomas R. Gaborski at the Rochester Institute of Technology, Rochester, USA, investigating the role of pathogenic bacterial extracellular vesicles in sepsis and in disruption of the blood-brain barrier.


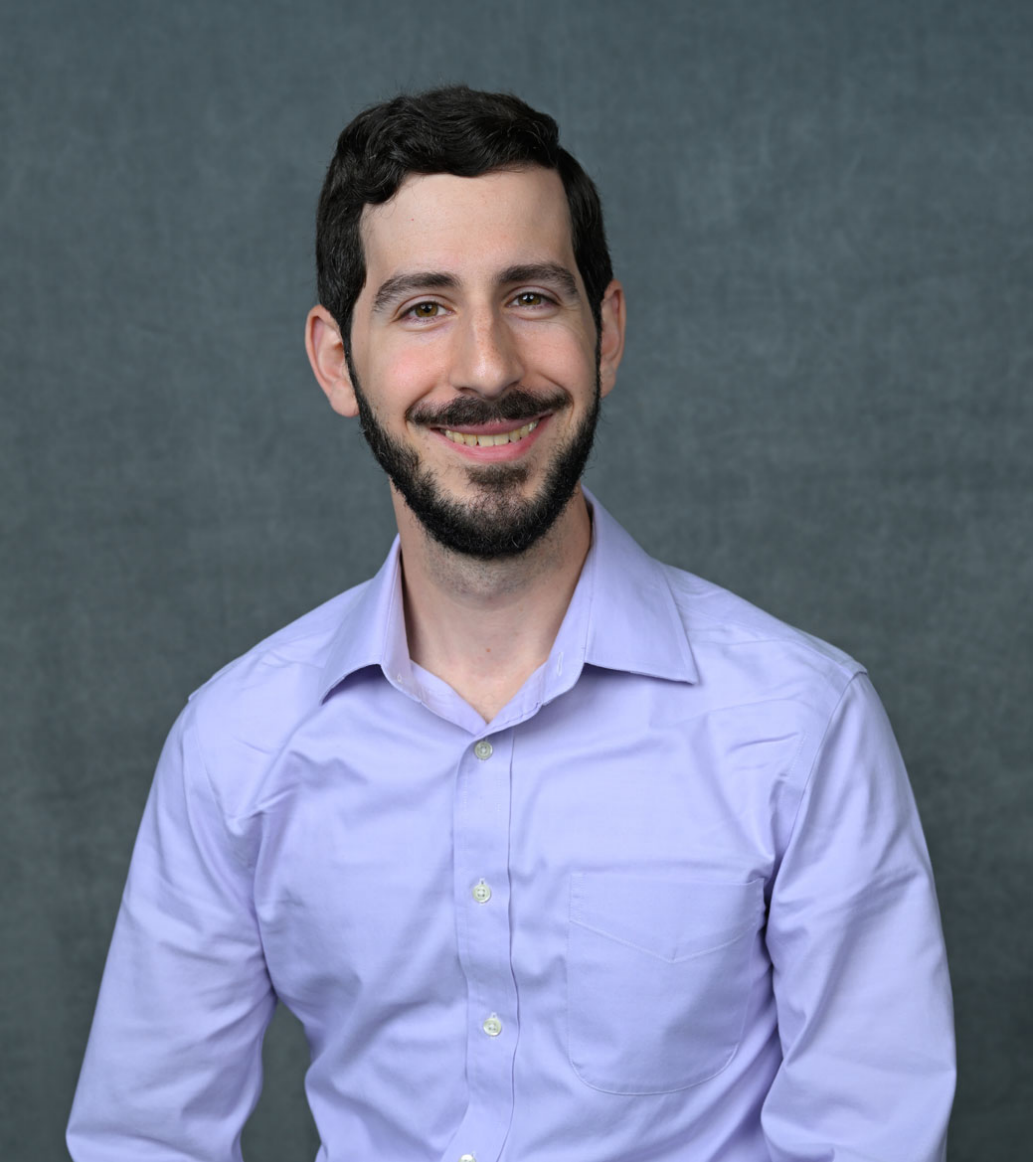


**Louis Widom**

**Describe your scientific journey and your current research focus**

In my graduate training, I first focused on validating a lab-on-chip model of the blood-brain barrier by examining deposition of extracellular matrix proteins. That initial work expanded into studies of endothelial function under pro-inflammatory conditions. I did not originally expect to be working with bacterial extracellular vesicles, but they quickly became central to my PhD research. At the time of this writing, I am preparing to defend my thesis and transition to the next phase of my scientific career.

**Who or what inspired you to become a scientist?**

Both of my parents have biology and biochemistry training, so I was exposed to these topics from a young age. In addition, I credit my high school biology teacher as well as various science fiction television programs and films for inspiring me to pursue scientific study. After obtaining my bachelor's degree, I worked for a few years in the Regeneron Genetics Center and was further motivated to apply to graduate programs to obtain the training necessary to become an independent researcher.

**How would you explain the main finding of your paper?**

Bacteria produce nanoparticles that cause inflammation. We demonstrated that treating bacteria with different types of antibiotics leads to changes in the potency of these nanoparticles, which can alter the response of the cells that line blood vessels. We identified some of the stimulatory cargo in the nanoparticles that are modified by the antibiotic treatments and may explain the measured differences in potency.

…if we can inhibit pro-inflammatory stimulation caused by this cargo, we believe that this will improve patient survival rates from diseases such as sepsis

**What are the potential implications of this finding for your field of research?**

Antibiotic treatment is important for eliminating bacterial infections but can change the pro-inflammatory characteristics of the nanoparticles produced by bacteria. To improve outcomes for patients undergoing treatment for severe infections, it is necessary to develop new anti-inflammatory medications. We hypothesise that some of the cargo that we identified in bacteria-produced nanoparticles could be good therapeutic targets to pursue. In the future, if we can inhibit pro-inflammatory stimulation caused by this cargo, we believe that this will improve patient survival rates from diseases such as sepsis.

**Figure:**
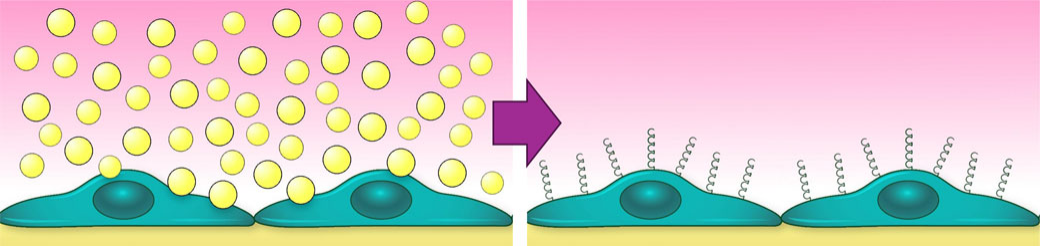
Illustration of endothelial cells increasing ICAM-1 on their surfaces in response bacterial extracellular vesicle treatment.

**Which part of this research project was the most rewarding?**

Prior to this project, I only had a passing familiarity with microbial research. I learned a lot during this study and had the opportunity to present some of our results at the 2025 American Society of Microbiology (ASM) meeting. The field is unsurprisingly vast, though it seems that the topic of pathogenic bacterial extracellular vesicles is still relatively niche. As such, it has been rewarding to expand knowledge within this sub-community.

**What do you enjoy most about being an early-career researcher?**

It is a cliché response, but I most enjoy the people with whom I have had the pleasure to work. Graduate research can proceed for long, seemingly fruitless stretches, so being surrounded by a supportive group is vital for maintaining sanity and a positive outlook.

**What piece of advice would you give to the next generation of researchers?**

Do not be afraid to pivot a research project if a particular direction or experiment repeatedly fails to work. Taking a new direction can often prove successful. On that note, side projects are worth pursuing if you have the time and energy.

**What's next for you?**

As mentioned above, I am currently wrapping up my dissertation and should defend before March 2026. If anyone, particularly members of industry, has positions available for an expert in extracellular vesicle isolation, vascular modelling, lab-on-chip devices, microscopy, and image analysis, please do not hesitate to reach out.
